# Assessing Food Preferences in Dogs and Cats: A Review of the Current Methods

**DOI:** 10.3390/ani5010126

**Published:** 2015-03-18

**Authors:** Christelle Tobie, Franck Péron, Claire Larose

**Affiliations:** SPF Diana, ZA du Gohélis, Elven 56250, France; E-Mails: fperon@diana-petfood.com (F.P.); cforges@diana-petfood.com (C.L.)

**Keywords:** pet food, palatability, acceptance, preference, dogs, cats, emotional palatability, pet parenting, cognition

## Abstract

**Simple Summary:**

The objective of this review is to present the different approaches and techniques used to assess petfood palatability, either with expert panels or naïve individuals (in-home panels).

**Abstract:**

Food is a major aspect of pet care; therefore, ensuring that pet foods are not only healthful but attractive to companion animals and their owners is essential. The petfood market remains active and requires ongoing evaluation of the adaptation and efficiency of the new products. Palatability—foods’ characteristics enticing animals and leading them to consumption—is therefore a key element to look at. Based on the type of information needed, different pet populations (expert or naïve) can be tested to access their preference and acceptance for different food products. Classical techniques are the one-bowl and two-bowl tests, but complementary (*i.e*., operant conditioning) and novel (*i.e*., exploratory behavior) approaches are available to gather more information on the evaluation of petfood palatability.

## 1. Introduction

The petfood market remains active and dynamic. Recent data revealed a constant increase in the pet population reaching 3.5 billion dogs and cats worldwide in 2014 [1]. In parallel, pet food sales are increasing even faster reaching 131.7 billion euro [2] over the same period. The petfood industries are regularly innovating and developing new products/formulas. Between January 2013 and October 2014 more than 4000 snack and 6000 food products (3000 dry and 3200 wet pet foods [3]) were launched on the market worldwide.

When developing new products, the petfood industries have to find a compromise between nutritional quality and palatability, particularly for diets claiming health benefits such as obesity or diabetes management. Even the best formulated diets can be inefficient or not popular among pet owners if the animal refuses to eat it. Palatability is consequently a crucial attribute for pet foods.

The hedonic properties of food are often defined by an attractive taste and are understood not only through the sensory characterization of food, such as smell, taste and mouthfeel but also through the nutritional and physiological post-ingestion effects. Palatability is related to how readily a food is accepted and measured in terms of its attractiveness and consumption. Because understanding animals’ preference is not obvious, indirect objective methodologies have to be developed in order to rank different products based on animal feeding behaviors and reactions. Assessment of palatability in companion animals is strategic for developing foods, treats and (oral) medications that they will consume.

Domestic dogs and cats have different nutritional requirements [4], feeding behaviors [5,6] and are sensitive to numerous palatability drivers [7]. The sensory analysis of diets by pets is mostly based on preference and/or acceptance tests [8]. Such trials can be performed in in-home panels of naïve pets and in expert panels of animals trained to discriminate foods with different nutritional and sensory properties since they were young. In preference testing, animals have the choice between two different diets presented simultaneously whereas in the acceptability tests, only one type of food product is available. To enrich the classical palatability measurements, new methods and criteria have been developed focusing on selected animal behaviors proven to reflect in an innovative way, pet foods’ palatability performance.

Feeding pets or giving them treats is a key moment which strengthens the bond between the owners and their animals. Referring to emotions and perceived palatability, recent protocol developments also took in consideration owners’ perception of their pets’ feeding enjoyment and consequently their perception of a diet’s palatability. The important point is that all these palatability measurement methods are complementary; they can be combined to finally deliver an exhaustive evaluation of pet foods’ overall palatability and performance.

In this review, the pros and cons of the different classical methods to assess petfood palatability are presented as well as newly developed and complementary techniques.

## 2. Panels and Methods Classically Used to Assess Food Preference and Acceptability

Palatability assessment tests can be run on two types of animal panels: either in pet centers with expert panels or in an in-home environment with owner’s pets. Both approaches have advantages and constraints [9]. During the product development stage, the scientific and technical questions will lead towards one or the other option. Expert panels perform palatability tests on a daily basis. They can be specialized on one type of food (dry only or wet only) or test different types of diets with transition periods between each. The expert pets are more reliable and accurate than in-home pets, but need intensive training to be exposed to a diversity of foods; qualification tests when one product is known to be highly palatable compared to the other or when products are known to be equal to check that animals select as expected; and a permanent quality follow up to check their accuracy in discrimination, reproducibility of answers, and potential lateralization [10].

**Figure 1:**
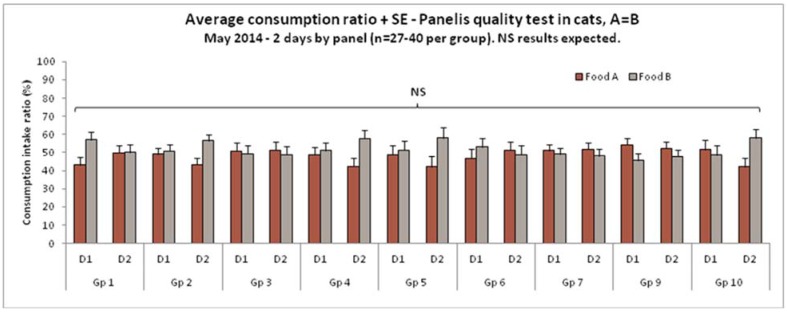
Average consumption ratio and Panelis quality test in cats, A = B, May 2014, 2 days by panel (*n* = 27–40 per group). None significant (NS) results expected.

Quality tests should be conducted regularly in expert panels to control for any side bias. In this type of test, the animals are offered the same food and the expected outcome is to observe no significant difference (see Figure 1).

The repeatability of testing conditions and the control of environmental perturbations are also among the key-characteristics of expert panels. To build such supervised “samples” of expert animals and to analyze this type of data (bimodal distribution), a minimum number of 30 individuals is necessary to secure statistical robustness. This level of requirements and control, on the dietary past for example, are necessary to limit the impact of biases (novelty effect, panel effect, *etc*.) on answers and obtain significant results even on finer differences [10]. In order to secure the reliability and relevancy of results, palatability assessment should also be performed on pets of varied ages, sizes and breeds, in good veterinary conditions and even more, on pets undergoing no stress.

In-home panels are constituted of family-owned pets that are selected according to different criteria (age, sex, dietary history, *etc*.). These tasters are naïve and do not have any training. They also have a lower testing frequency than expert panels and testing conditions are less controlled. In comparison with expert panels in-home pets feeding history can be vague and can lack diversity. It is very difficult to make sure that the testing protocol has been respected and that the owners’ perceptions have not biased objective measurements. For all these reasons, palatability tests performed in such panels should include a lot more animals: ideally ≈100. The automation of the data collection can also provide additional reliability for the quantitative data gathered [11]. On the other hand, the main advantage of an in-home panel is in providing data representative of the final market: to get “real-life” feedback. Furthermore it is a good way to evaluate owners’ reactions to the products’ cosmetics and about their perception of palatability through pet-centric criteria.

**Figure 2:**
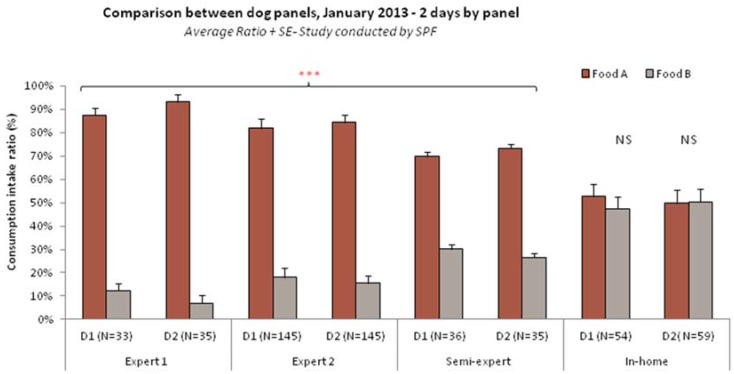
Comparison between dog panels, January 2013, 2 days by panel. Average Ratio + SE, Study conducted by Diana Pet Food.

The comparison between in-home and expert panel results often reveals differences, more or less important according to the type of product tested. Semi-expert panels consist in in-home dogs and cats trained and qualified to perform preference tests. For example, two-bowl tests performed on expert, semi-expert and in-home panels comparing different commercial dog dry foods showed that the outcome and conclusion could vary according to the panel used (see Figure 2).

For example in their study, Griffin *et al.* [9] found more consistency between the different panels when testing wet products. It was also noted that in-home panels may be more stable in their preferences [9] but that expert panels may be better discriminating small differences. Such discrepancies can be explained by all the factors we have just listed, related to pets’ level of training, to their feeding history and to the testing environment.

Referring to the testing protocols, different approaches can be used in order to assess palatability differences between pet foods. In general all classical methods are based on the amount of each food consumed during a definite period. Two protocols are most commonly used. Preference testing is based on a simultaneous presentation of two diets, in order to measure if a preference is expressed by pets through quantities eaten of each product. Acceptance testing consists in the presentation of one diet only and in the assessment of the quantity eaten, as the expression of the product’s intrinsic palatability. The food can be available for a limited period (for dogs for example) or for a longer period or even *ad libitum* to reproduce the “natural” conditions encountered in the home environment or, to respect a more natural feeding rhythm, for cats particularly.

The two-bowl test (or paired stimulus or *versus* test) compares how much of two foods, presented simultaneously, is eaten in a defined period of time. This is the most common test used in expert panels for dog and cat palatability assessment studies. It compares two products and establishes a preference based on the difference of quantities consumed. Waterhouse and Fritsch [12] and Hegsted *et al*. [13] described the general method and the possible factors that could influence the results. In such tests, two identical bowls are delivered simultaneously to the tested animal, each bowl containing one of the two products to be tested (A or B). The animal has free access to the bowls for a preset period of time. The quantity available in each bowl is more than sufficient to cover the energetic requirements. In general, the test is conducted in individual enclosures to avoid any social interactions or competition. At the end of the feeding time or when one bowl is finished, bowls are taken back and weighted again to measure the quantity consumed. For each pair of products tested a second test maybe necessary, this time switching their relative position, in order to control any position bias. This second measure enables an evaluation of repeatability.

Important parameters in this two-pan test include the first choice that is the first food product tasted, reflecting the olfactory perception and attractiveness; the amount of food consumed; the ratio (A/B) of food consumed; the percentage of food intake (A/(A+B)) [14] and the preference ratio (quantity of food A consumed over the total of food distributed). Usually the percentage of consumption is used [15]. The two-pan test enables a ranking between different products but is not transitive as the “preference” is based on a forced choice. The palatability of the diet is not considered *per se* but in comparison to the other diet, which means that all the paired comparisons should be tested.

Some *versus* trials are conducted with pets in-home [11], but it is generally less precise in this condition due to the lack of environment control (for example greedy dogs finishing both bowls if the owner did not remove them early, or several cats sharing the same bowl, *etc*.). Thus, it is preferable that the two-bowl tests be conducted using expert panels as they allow control of the possible bias [10,12,16].

One inconvenience of two-pan testing is that the method does not offer control of how different foods (smell and taste) may affect the palatability of each other or of the long-term effects of caloric and nutritional value. This technique may also lead to animals consuming excessive quantities of food, if necessary human resources are not available to remove bowls when one is finished, or enough food from both bowls is consumed.

The one-bowl test (or one-pan, or single stimulus, or monadic test) [17], in which the animal has free access to a single food for a determined amount of time, is used to measure only the acceptability of a food product. This method is quite similar to the situation that can be found at home where a pet-owner introduces a new food product [18] and, thus, is well adapted to in-home panels. The indicators of this kind of testing will be mostly the quantity consumed and sometimes the speed of consumption. Furthermore, when tested in-home, it is possible to use questionnaires in order to enrich the information gathered. For example, additional data such as human perceptions of the food [19] or the animals’ enthusiasm to eat it [11,20] can be collected. Thus, owners can provide not only information about home environment and dietary history of their animal but also their impressions on the different diets and report any behavior or physiological modifications.

Several factors can influence the results of the one-bowl test such as the seasonal effect (*i.e*., in cats: eating less during winter [21]) or a daily variation (*i.e*., dog eating more during their afternoon meal compared to the morning meal; personal communication) requiring a calculation of a reference consumption level and adapted distributed ration that would take into account those factors. This is particularly important as the interpretation of the “unique” value delivered may sometimes be complex.

## 3. Complementary Methods and New Approaches

It is possible to implement complementary indicators and provide additional information to classical preference and acceptance tests.

### 3.1. Liking Test

**Figure 3:**
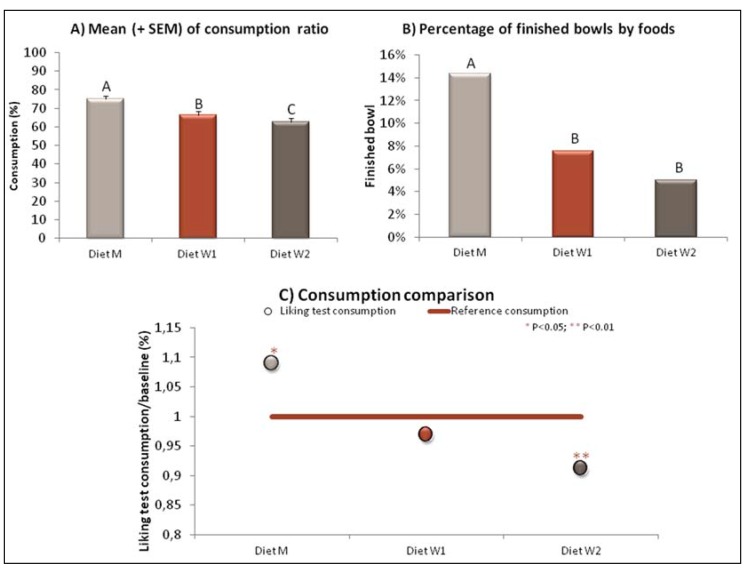
Evaluation of diets M, W1 and W2 by a Liking test conducted on an expert panel (*n* = 38, Panelis) (**A**) consumption ratio, (**B**) finished bowls and (**C**) consumption of the diets expressed relative to a reference consumption. Differing letters identify significant differences between the products.

The Liking test consists of a one-bowl test with adjusted food quantities, which enables the animal to finish the bowl infrequently, and available for a preset period of time [22]. The monitored indicators reflect meaningful criteria for owners in the understanding of their pets’ feeding enjoyment: the percentage of finished bowls and refusals; the consumption speed; and the gap with the reference consumption rate (RCR) of each individual (see Figure 3). The RCR consists of the ratio between the individual level of consumption at the test and the individual reference consumption based on its food intake history and other natural variations (season, meal of the day, *etc.*). The analysis of the deviation is reported for the all panel. If the difference is significant and negative: individuals of the panel did not like the food (or at least eat less than usual) and on the contrary if the difference is significant and positive, individuals enjoyed their food.

This one-bowl test is run with expert panels as it was developed to provide higher accuracy and reliability thanks to the control of specific biases potentially impacting monadic testing at home. It is even possible to rank different products tested with monadic tests but some parameters should be adapted, such as the randomization of the order of the different food products presented across the tested population and specific statistical treatment. A mixed model is used to analyze the differences between groups’ means on fixed variables. A random variable is used to take into account the individual variability and to extrapolate the results to a larger population.

### 3.2. Kinetics

**Figure 4:**
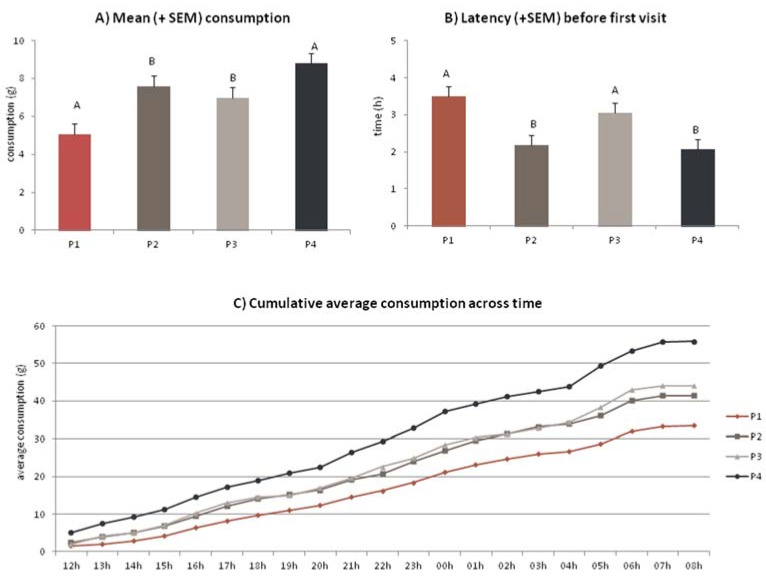
Four dry cat foods, differing only by the composition of the palatability enhancer applied in coating, were evaluated in sequential monadic conditions by a 40 cats expert panel (Panelis). Individual data such as time and quantities eaten were recorded automatically during a 20 h period. (**A**) average consumption per passage (adjusted mean +SEM); (**B**) average time before the first visit (adjusted mean + SEM); and (**C**) cumulative average consumption across time. Differing letters identify significant differences between the products (*p* < 0.05).

Fine-grained measurement is the cumulative amount eaten on a moment-by-moment basis during the test used both for one-bowl and two-bowl tests. This approach can provide quantitative information about individual feeding styles (rapid eaters *vs.* slow eaters), the way animals distribute their feeding between two foods in a choice test and, in some cases, the initial disruptive effects of a new diet [18]. Using the one-bowl method on a preset period of 20 h with a follow up of the quantity consumed helps to compare the profile of acceptance of the different products [23], which did not distinguish one from the other during the preference test. Kinetics can be used to measure new indicators of performance and enjoyment, including criteria reflecting attractiveness: average time before the first visit (passage or feeding events), average consumption per feeding events, number of passage without consumption, *etc.* (see Figure 4).

This tool enables to differentiate between an immediate attractiveness and its evolution across time. It is also adapted to the observation of feeding behaviors in front of specific diets: individuals—cats particularly- are supposed to reduce their number of visits to the bowl and/or meal size when fed with a “satiety” product compared to a standard food product. At the end of the feeding period we may observe diets consumed in different quantities, the consumption rate of the “satiety” product being slower. Even over a medium-term food intake study, dogs showed a difference in their feeding pattern with a satiety effect more pronounced for the high-protein high-fiber diet compared to the high-protein low-fiber diet, with both diets having equivalent palatability [24].

In preference testing (two-bowl test) and in acceptability testing (one-bowl test) the animal performing the test may not have other food choices than the one presented, and may be forced to eat a food even if they find it less palatable. Furthermore the post ingestion effects can impact on the expression of a preference, for instance, when testing weight management diets where it would be difficult to distinguish enhanced efficacy and reduced palatability. Complementary methods can be used looking not only at the quantity eaten but also at the “value” of the food when use as a reward in a learning task.

### 3.3. Concurrent Schedule Paradigm (CSP)

The concurrent schedule paradigm or operant conditioning is a procedure used to assess the strength of an animal’s motivation to eat [25]. This method used to compare quantitative and qualitative differences (quantify the hedonic value of foods) requires a specific motor action directed to specifically designed device such as a lever-pressing apparatus [25,26] or through human-animal interaction (e.g., nose-touching on experimenter’s hand; [27]). This kind of approach assesses animal’s reactivity to food with minimum post-ingestion complications (relatively small food quantity) and enables comparison of very different food products as it is the “currency” (*i.e*., motivation to press the lever) that is compared. Technically demanding as it may requires a specific device and software and time consuming, the CSP requires highly trained individuals, tested over a relatively long period.

### 3.4. Cognitive Palatability Assessment Protocol (CPAP)

Araujo and Milgram [28] developed a method based on associative learning consisting in an object discrimination learning task where the animal can express a preference without any food intake. The dog can interact with three different objects, two of them paired with different types of food. Once the animal develops a preference for a specific object and its associated outcome, the pairs are switched and the dog has to learn the new association if he wants to keep receiving its “preferred” food product. The no reward object provides a control for individuals without any preference between the two compared foods. This preference testing approach can vary to examine short- or long-term preferences without confounding nutritional or caloric effects. While comparing the CPAP with the two-pan tests, the authors found that it was possible to compare different types of products (moist *vs.* dry for instance); that the differences between the products were stronger even with small sample size; and that the preference was stable across time and repetitions [29]. The results suggest that the CPAP is less sensitive to the effects of prior feeding and satiation than the two-bowl test [29]. This method also requires extensive training and more time but at the same time fewer individuals are necessary to obtain significant reliable data. The CPAP seems less adapted to cats as it requests sustained attention from the tested individual.

Concurrent schedule paradigm and CPAP methods may not be biologically relevant as they are very different from the real living conditions and feeding habits of the pets.

### 3.5. Exploratory Behavior

A previous experiment looked at the cat body language and behavior within a context of feeding period, with wet food, and found differences related to palatability [30]: licking or sniffing the food bowl and lip licking were associated with palatable food products whereas sniffing the food or nose licking reflected some aversion for the product. However, a similar study conducted on dry food did not reveal any difference (Personal communication). More recent studies assess differences in the perceived palatability of products using a spontaneous behavior: the olfactory exploration. The role of olfaction at feeding time is multiple [31]: to locate the food source—that may be less relevant for domestic species; to assess the food organoleptic aspects, toxicity, *etc.*; and to stimulate the gut secretion. Other studies have looked at the spontaneous behavior of the pets when facing different food products either presented sequentially (one-bowl test) or simultaneously (two-bowl test). In the Becques *et al*. [32] study for instance, the cats were video recorded during their feedings over several days. Behaviors and postures were coded according to different categories and correlated with food intake measures. In the dog study [33] individuals first had the opportunity to taste the different food products before experiencing the situation where a wire mesh was impeding the access to the different bowls. The authors looked at the time spent exploring both food locations. The results revealed that cats [32] spent significantly longer sniffing at the product less preferred. Dogs were the opposite [33] exploring longer the preferred food product. The difference between the two species could be explained by their natural history and dietary behavior [5,6] or by the difference in the protocol used, as cats could access the food product at the end, while dogs could not.

## 4. Conclusions

During the past 30 years, the same classical tests and criteria have been mostly used to assess pet food palatability. Only recently new approaches and complementary indicators have been developed in order to provide more information and enrich palatability measurement. Analyzed consumption parameters are no longer exclusively intake ratios, and new approaches are no longer exclusively focused on organoleptic and nutritional orientations; they are now considering the emotional dimension of palatability performance. New assessments integrate the triangular relationship in petfooding, considering not only the behavioral expressions of pets but also the interactions with their owners and finally the owners’ perception of their pet enjoyment.

Each of the methods counts advantages and disadvantages, but according to the type of questions, to the targets and the panel resources available, it is possible to select one or another method and sometimes even a combination of several. All the palatability measurement methods are complementary, can be combined and finally deliver an in-depth evaluation of pet foods’ comprehensive palatability and performance (see Table 1).

**Table 1:** Summary of the pros and cons of the different methods.

	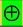 WHAT FOR	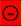 LIMITS	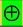 WHAT ELSE
**TWO-BOWL TESTS**	New products in development Measurement of fine differences (ingredient effects on same kibble base, screening of new formulas) Rather on expert panels	Forced comparison in expert panel ≠ in-home Not adapted to compare different nutritional values	CPAP to remove the influence of food intake Kinetics to differentiate palatability levels with new criteria and visualize how preference has been built
**ONE-PAN TESTS**	ON EXPERT PANELS:	Lack of accuracy due to uncontrolled parameters Possible subjective interpretation of owners	Kinetics to differentiate iso palatable products with new criteria or analyze consumption of different nutritional values or physiological effects Liking tests for new enjoyment and behavioral criteria
	Product development validation Measurement of product acceptability in natural conditions, of specific criteria related to enjoyment observation or to nutritional specificities such as weight management… Comparison of expected bigger differences		
	ON IN-HOME PANELS:		
	Access to owners perception		
